# Effects of Changes in the Levels of Damage-Associated Molecular Patterns Following Continuous Veno–Venous Hemofiltration Therapy on Outcomes in Acute Kidney Injury Patients With Sepsis

**DOI:** 10.3389/fimmu.2018.03052

**Published:** 2019-01-07

**Authors:** Jie Wu, Jianan Ren, Qinjie Liu, Qiongyuan Hu, Xiuwen Wu, Gefei Wang, Zhiwu Hong, Huajian Ren, Jieshou Li

**Affiliations:** ^1^Department of Surgery, Affiliated Jinling Hospital, Medical School of Nanjing University, Nanjing, China; ^2^Department of Surgery, Jinling Hospital, Nanjing Medical University, Nanjing, China

**Keywords:** continuous veno–venous hemofiltration, damage-associated molecular patterns, acute kidney injury, heat shock protein 70, high mobility group box 1, urinary nuclear DNA

## Abstract

**Background:** We investigated the association of damage-associated molecular pattern (DAMP) removal with mortality in sepsis patients undergoing continuous veno–venous hemofiltration (CVVH).

**Methods:** Circulating levels of DAMPs [mitochondrial DNA (mtDNA); nuclear DNA (nDNA); heat shock protein 70 (HSP70); and high mobility group box 1 (HMGB1)] and cytokines were measured at baseline, 6 and 12 h after initiation of CVVH. Urinary DNA levels were analyzed at baseline and end of CVVH. The expression of human leukocyte antigen (HLA)-DR was assayed at 0, 3, and 7 days after initiation of CVVH. Moreover, the effects of HSP70 and HMGB1 clearance on survival were analyzed.

**Results:** We evaluated 43 patients with acute kidney injury (AKI) (33 sepsis patients). Twenty-two sepsis patients (67%) and three non-sepsis patients (30%) expired (*P* = 0.046). Significant reductions in the levels of circulating interleukin-6 (*P* = 0.046) and tumor necrosis factor-α (*P* = 0.008) were found in the sepsis group. The levels of mtDNA were increased (*ND2, P* = 0.035; *D-loop, P* = 0.003), whereas that of HSP70 was reduced (*P* = 0.000) in all patients during the first 12 h. The levels of DAMPs in the plasma were markedly increased after blood passage from the inlet through the dialyzer in survivor sepsis patients. The clearance rates of HSP70 and HMGB1 were good predictors of mortality [area under the curve (AUC) = 0.937, *P* = 0.000; AUC = 0.90, *P* = 0.001, respectively]. The level of HLA-DR was increased in response to higher HSP70 clearance (*P* = 0.006). Survival was significantly worse in groups with higher clearance rates of HSP70 and HMGB1 than the cut-off value (log-rank test: *P* = 0.000 for both). Higher HSP70 clearance was a significant independent predictor of mortality (odds ratio = 1.025, 95% confidence interval [CI]: 1.012–1.039, *P* = 0.000). The urinary nDNA (β*-globin*) level before CVVH was an independent risk factor for the duration of CVVH in patients with sepsis (sRE = 0.460, 95% CI: 1.720–8.857, *P* = 0.005).

**Conclusion:** CVVH removes inflammatory factors, reduces urinary DAMPs, and removes plasma DAMPs. However, survival decreases in response to higher HSP70 clearance.

## Introduction

Sepsis is a life-threatening disease caused by a dysregulated host response to infection (1). In particular, acute kidney injury (AKI) is one of the most common types of organ dysfunction, appearing early in the course of sepsis. Nearly half of patients develop AKI in the intensive care unit and account for approximately half of sepsis-related deaths (2, 3). The high mortality associated with septic AKI may be at least partly explained by an incomplete understanding of its pathophysiology and a delay in diagnosis.

The levels of pro-inflammatory mediators in the serum are increased in patients with AKI, regardless of its cause (4, 5). Pro- and anti-inflammatory mediators, as well as damage-associated molecular patterns (DAMPs), play important roles in regulating the immunological response that mediates the severity and complications of sepsis (6, 7). DAMPs, also known as alarmins, are constitutively available endogenous molecules released in response to tissue damage and involved in the activation of the immune system. A subset of DAMPs are nuclear or cytosolic molecules, such as mitochondrial DNA (mtDNA), nuclear DNA (nDNA), high mobility group box 1 (HMGB1), and heat shock protein 70 (HSP70). In recent years, Rajaee et al. stated that the definition of interleukin (IL)-1 and IL-33 as DAMPs or cytokines remains controversial (8).

Recently, an increased level of mtDNA in the urine has emerged as a novel non-invasive biomarker for the detection of AKI (9). Moreover, our research has demonstrated the effectiveness of using urinary levels of mtDNA as evidence of renal mitochondrial injury induced by AKI after sepsis (10). However, the role of circulating DAMPs in AKI is controversial (11, 12).

Currently, continuous renal replacement therapy (CRRT) is the main support strategy for AKI, involving several types of treatment. Considerable evidence has suggested that CRRT controls azotemia and fluid balance (13, 14). It has been reported that continuous veno–venous hemofiltration (CVVH) may assist in reducing acute inflammation through the removal of pro-inflammatory cytokines and signaling molecules, such as the tumor necrosis factor (TNF)-α and various ILs (4, 15). However, the effectiveness of pro-inflammatory molecule clearance is controversial. Several clinical studies have shown that changes in the type of CRRT modes did not reduce mortality compared with the standard mode, even after extensive removal of pro-inflammatory cytokines in the new mode groups (16, 17).

Studies investigating DAMPs and CRRT are limited to a particular molecule and the relationship between levels of DAMPs in the plasma and complications at a certain point in time (18). Thus far, there is no study reporting the effect of CVVH on the levels of DAMPs and the effects of these changes on the prognosis of patients. Therefore, the present study investigated the effects of CVVH on the circulating and urinary levels of DAMPs in AKI patients and the roles of DAMP clearance on patient outcome.

## Materials and Methods

### Study Population

The population of this prospective study consisted of 43 patients with AKI requiring CVVH who were admitted to the surgical intensive care unit (SICU) of Jinling Hospital (Nanjing, China). The study was approved by the Ethics Committee of Jinling Hospital, Nanjing and conducted from November 2016–August 2017. Written informed consent was provided by all enrolled patients prior to their participation in the study.

The inclusion criteria were: age >18 years; presence of AKI requiring CVVH; presence of sepsis. The exclusion criteria were: refusal to provide consent; history of chronic kidney disease; expected duration of CVVH <12 h; death within 24 h from initiation of CVVH; ongoing CRRT; and previous renal transplantation. Baseline demographic and clinical data were automatically recorded using a software (Nanjing Haitai Medical Information System, Nanjing, China) or by physicians. For comparison, 10 age- or sex- matched AKI patients without sepsis receiving CVVH were included.

### Definitions

We defined AKI according to the Kidney Disease: Improving Global Outcomes (KDIGO) criteria (19), corresponding to stage 1 of the KDIGO classification with increased serum creatinine level ≥0.3 mg/dL (≥26.5 μmol/L) within 48 h or increased serum creatinine ≥1.5-fold compared with baseline within 7 days. Sepsis and septic shock were defined according to the Third International Consensus Definitions for Sepsis and Septic Shock (1). Indication of CVVH required at least one of the following criteria: oliguria (urine output <100 mL continued for 6 h after adequate fluid resuscitation), serum creatinine (Scr) >250 μmol/L (2.8 mg/dL), serum potassium concentration >6.5 mmol/L, severe acidemia (pH < 7.2), and presence of severe fluid overload.

### CVVH Procedure

All patients were treated with CVVH using the Aquarius hemodialysis system (Baxter International Inc., Chicago, IL, USA). Central venous catheterizations (Quinton-Mahurkar dual-lumen hemodialysis catheters, Kendall, Tyco Healthcare Group LP, USA) through femoral vein sites were used for vascular access. Administration of CVVH was similar for all patients under the following parameters: blood flow, 100–200 mL/minnum; filter, high flux AV600S (polysulfone, 1.4 m^2^, Fresenius Medical Care); replacement fluid infused at 4 L/h through the pre-dilution route. The daily net ultrafiltration volume was decided by the attending physician. Safety monitoring, including serum electrolyte balance, acid base status, and fluid balance was performed twice daily. Anticoagulation treatment was performed according to the patient's condition. In general, citrate anticoagulation was combined with low-dose heparin to maintain the activated clotting time within the extracorporeal circuit within a desired range (200–250 s). In addition, mechanical ventilation, vasopressor therapy, or any other standard treatment was used regardless of the presence of sepsis, as long as the treatment indications were met.

### Sample Collection and Processing

Collection of blood samples (12 mL) was performed from both the inlet and outlet of the dialyzer at baseline and 6 and 12 h after initiation of CVVH. Of this sample volume, 4 mL were collected in ethylenediamine tetracetate tubes, whereas the remaining 8 mL were stored in promoting coagulating tubes. Ultrafiltrate collections (10 mL) were drawn within the first 2 h. Urine samples were collected at baseline and end of CVVH. All samples were immediately transported to the laboratory and placed on ice. Blood samples were centrifuged at 3,000 × g at 4°C for 10 min, whereas the ultrafiltrate collections and urine samples were centrifuged at 1,000 × g at 4°C for 5 min. Supernatants were collected and stored at −80°C until further analysis. Samples were collected in the first 12 h of CVVH.

### Isolation and Quantification of mtDNA and nDNA

Free DNA was isolated from 200 μL plasma samples using the QIAamp DNA Blood Mini Kit (Qiagen, Valencia, CA, USA), and 1.75 mL urine samples using an urine DNA isolation kit (NorgenBiotek, Ontario, Canada), DNA was eluted in 100 μL of supplied buffer as previously described (10, 20). Quantitative polymerase chain reaction [qPCR; real-time polymerase chain reaction (PCR)] targeting mitochondrial genes *(D-loop* and *ND2*) and nuclear genes (*GAPDH* and β*-globin*) was performed to quantify cell-free DNA (cf-DNA) content. The efficiency of all reactions was >98%. All samples were analyzed in triplicate. The standard curve of the quantitative assay was produced through the serially diluted template cloned into a plasmid DNA.

### Analysis of Cytokines and DAMPs

The different cytokines (IL-1β, IL-6, TNF-α, IFN-γ, IL-10) and HSP70 and urinary neutrophil gelatinase-associated lipocalin (NGAL) were quantified using an enzyme-linked immunosorbent assay (ELISA) kit (R&D System, USA), while the levels of HMGB1 were quantified using a different ELISA kit (SAB, USA), according to the instructions provided by the manufacturer.

Based on the mass conservation principle, the removal rate of cytokines during CVVH was calculated as follows (without considering the concentration of cytokines at baseline):

Ctr = (Ci–Co)/Ci
Clr = Ctr × Qb
Ctr, Total concentration removal rate
Ci, Concentration in the inlet plasma prior to the addition of replacement fluid
Co, Concentration in the outlet plasma
Clr, Clearance rate
Qb, Inlet blood flow rate (mL/min).

### Quantification of Monocyte Human Leukocyte Antigen-DR (mHLA-DR)

Quantification of mHLA-DR was performed according to the description by Döcke et al. (21). In brief, whole blood was acquired at 0, 3, and 7 days after initiation of CVVH and lysed using red blood cell lysis buffer (KeyGEN BioTECH, Jiangsu, China). Subsequently, they were fixed in 4% paraformaldehyde and incubated with Anti-HLA-DR/Anti-Monocyte Stain (Becton Dickinson, San Jose, CA, USA). Samples were analyzed using a FACScan (Becton Dickinson, San Jose, CA, USA) with a five-color upgrade (CyTech, Fremont, CA, USA). Flow files were analyzed in CellQuest Pro (Becton Dickinson, San Jose, CA, USA). Antibodies bound per cell (ABC) were calculated by standardizing HLA-DR geometric mean fluorescence intensity (GMFI) of monocytes to BD Quantibrite-phycoerythrin (PE) beads (Becton Dickinson, San Jose, CA, USA).

### Statistical Analyses

Results are expressed as the mean ± standard deviation (SD) or median with interquartile range (IQR), as appropriate. Comparison of continuous variables between the two groups was conducted using the Student's *t*-test or Mann–Whitney *U*-test depending on Gaussian distribution. Categorical variables were compared between the two groups using the chi-square test and Fisher's exact test, as appropriate. We analyzed all datasets using a two-way repeated-measure ANOVA to examine the effects of CVVH duration. Analyses of receiver operating characteristic (ROC) curves were conducted to test the effectiveness for the prediction of certain outcomes. The optimal cut-off value was defined as the value closest to the Youden Index. Correlation analyses were performed on multiple variables, and the degree of correlation was determined by calculation of the Spearman rank-order coefficients. The Kaplan–Meier method and binary logistic regression analysis was used to determine predictors of mortality. Linear logistic regression analysis was performed to determine independent predictors of CVVH duration. The criterion for statistical significance in all comparisons was *P* < 0.05. All analyses were performed using the SPSS v21.0 software (IBM, Armonk, NY, USA).

## Results

### Demographic and Outcome Parameters of AKI Patients With or Without Sepsis

**Figure 1:**
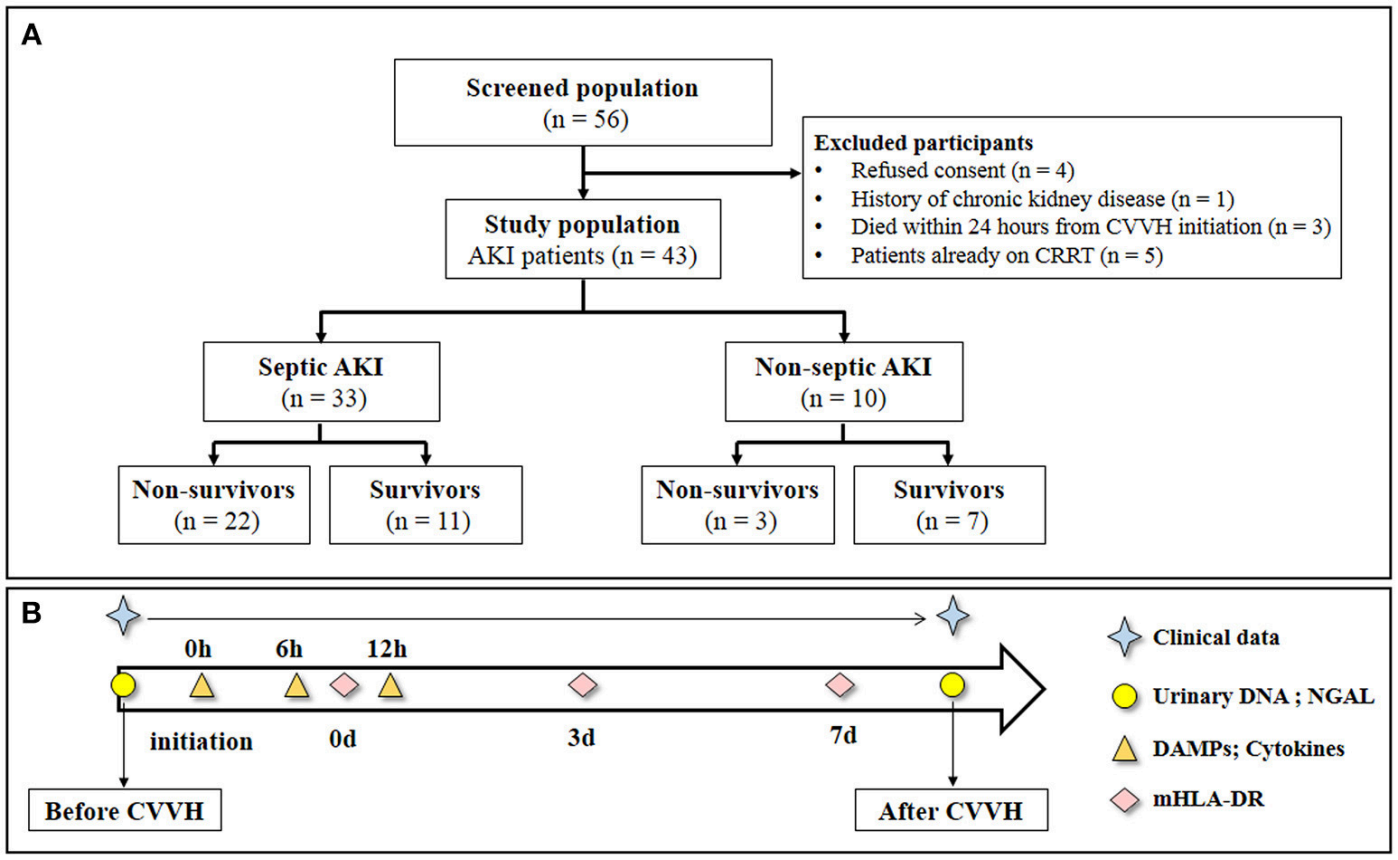
Study flow and schedule for samples collection. **(A)** Study flow. **(B)** Clinical data was obtained every day during the study period. Urinary DNA (mitochondrial DNA and nuclear DNA) and urinary NGAL were measured at baseline and end of CVVH. The level of Circulatory cytokines and DAMPs were measured from the inlet and outlet of the dialyzer at baseline, 6 and 12 h after CVVH initiation. Levels of mHLA-DR were measured at 0, 3, and 7 days after CVVH initiation. CVVH, continuous veno-venous hemofiltration; NGAL, neutrophil gelatinase-associated lipocalin; DAMPs, damage-associated molecular patterns; mHLA-DR, mononuclear human leukocyte antigen-DR.

The study flowchart is shown in Figure 1. The samples and clinical data were collected at the specified time points as shown in Figure 1B. The consecutive case series included AKI patients who met the criteria for CVVH indication, excluding those with chronic kidney disease. According to the hospital records and laboratory examination, the patients were classified as sepsis-associated AKI or non-sepsis-associated AKI. A total of 43 patients were enrolled in the study. Among them, 33 patients presented with sepsis, whereas 10 AKI patients presented without sepsis (control group). The aim of this classification was to investigate differences in the clearance rate of cytokines or DAMPs during CVVH between different diseases. All patients conformed to the criteria of CVVH and the characteristics of patients are shown in Table 1.

**Table 1:** Clinical characteristics and biochemical variables of the study population.

**Characteristics**	**CVVH patients**			***P-*value**
	**Total (*n* = 43)**	**Sepsis (*n* = 33)**	**Non-sepsis (*n* = 10)**	
**DEMOGRAPHIC DATA**				
Age, mean (SD), y	46.5 ± 13.8	48.9 ± 13.9	37.3 ± 9.4	**0.046**
Male, *n* (%)	31 (72.1)	23 (69.7)	8 (80.0)	0.642
BMI, mean (SD)	25.7 ± 4.3	25.3 ± 4.0	49.0 ± 14.0	0.237
SOFA score, mean (SD)	10.6 ± 5.1	11.3 ± 5.2	8.0 ± 4.1	0.132
APACHE II score, mean (SD)	13.5 ± 6.4	14.5 ± 6.2	9.9 ± 6.0	0.089
**CVVH PARAMETERS**				
Duration of CVVH, median (IQR), d	6.0 [3.5–13.0]	8.0 [2.8–19.3]	5.0 [4.0–7.0]	**0.046**
Blood flow, median (IQR), mL/min	140 [120–160]	155.0 [120.0–165.0]	140 [130–160]	0.071
Replacement fluid dose, median (IQR), mL/h	130 [130–145]	130.0 [130.0–142.5]	145 [130–260]	0.357
Observed effluent rate, mL/h	4,000	4,000	4,000	
**ICU ADMISSION**				
**Biochemical parameters**				
24h Urine output, median (IQR), ml	470 [91–1013]	280.5 [87.5–925.0]	750.0 [348.0–1740.0]	**0.009**
Creatinine, mean (SD),μmol/L	339.3 ± 252.5	323.7 ± 220.0	397.1 ± 365.2	0.503
BUN, mean (SD), mmol/L	20.1 ± 14.2	18.9 ± 10.6	24.9 ± 3.9	0.321
eGFR, median (IQR), mL/min/1.73 m^2^	17.8 [11.1–52.9]	16.9 [11.1–49.1]	21.0 [5.2–59.8]	0.418
Urinary NGAL, median (IQR), ng/mL	8.9 [4.7–10.6]	9.4 [6.0–10.5]	4.5 [0.4–16.3]	0.750
ALT, median (IQR), U/L	47.0 [22.0–84.0]	55.0 [23.0–89.0]	32.0 [19.0–69.0]	0.186
AST, median (IQR), U/L	68.5 [37.8–152.0]	67.0 [39.5–55.0]	70.0 [33.0–89.0]	**0.022**
CRP, mean (SD), mg/L	194.6 ± 92.3	191.4 ± 96.3	206.5 ± 81.1	0.705
PCT, median (IQR), ng/mL	8.8 [2.8–26.3]	7.8 [2.8–24.5]	14.4 [2.8–70.3]	0.232
Leukocyte count, mean (SD), × 10^9^/L	13.8 ± 7.4	13.38 ± 7.23	15.24 ± 8.65	0.565
RBC, mean (SD), × 10^9^/L	3.5 ± 1.1	3.37 ± 1.12	3.85 ± 1.06	0.312
PLT count, median (IQR), × 10^9^/L	129.0 [86.5–288.0]	134.5 [87.3–224.5]	117.0 [67.0–239.0]	0.163
APTT, mean (SD), s	42.4 ± 20.5	43.7 ± 22.3	37.6 ± 11.5	0.492
PT, mean (SD), s	15.3 ± 1.7	15.2 ± 1.6	15.5 ± 2.1	0.650
INR, median (IQR)	1.3 [1.2–1.5]	1.3 [1.2–1.5]	1.4 [1.2–1.5]	0.653
Serum albumin, mean (SD), g/L	56.7 ± 12.0	29.0 ± 5.0	33.1 ± 8.6	0.111
Total Bilirubin, median (IQR), μmol/L	34.0 [24.3–121.0]	32.5 [23.7–114.3]	37.9 [24.1–135.9]	0.055
Serum sodium, mean (SD), mmol/L	139.4 ± 6.1	139.8 ± 5.3	137.7 ± 8.6	0.439
Serum potassium, mean (SD), mmol/L	4.7 ± 1.0	4.7 ± 1.1	4.4 ± 0.8	0.476
Serum calcium, mean (SD), mmol/L	1.9 ± 0.5	1.9 ± 0.5	2.0 ± 0.3	0.776
Serum phosphorus, mean (SD), mmol/L	1.5 ± 0.8	1.5 ± 0.7	1.5 ± 1.1	0.983
**Serum cytokine levels**				
IL-1b, median (IQR), pg/mL	14.9 [7.9–18.3]	15.3 [8.4–19.7]	11.3 [3.0–16.7]	0.199
IL-6, median (IQR), pg/mL	72.9 [17.5–142.7]	95.6 [17.6–191.7]	33.9 [10.0–79.8]	0.503
IFN-γ, median (IQR), pg/mL	149.4 [79.0–272.8]	203.6 [99.7–268.8]	80.9 [27.1–445.6]	0.423
TNF-α, median (IQR), pg/mL	17.9 [4.5–155.6]	16.2 [4.6–112.8]	54.4 [2.1–205.6]	1.000
IL-10, median (IQR), pg/mL	4.2 [1.2–13.7]	4.3 [1.2–13.1]	4.2 [0.9–15.9]	0.682
**Serum or plasma DAMPs levels**				
HSP70, median (IQR), ng/mL	34.4 [14.2–124.4]	34.7 [14.7–128.2]	32.6 [9.6–129.6]	0.110
HMGB1, median (IQR), pg/mL	838.5 [666.2–1013.1]	836.1[653.8–1001.8]	902.0 [678.7–1160.9]	0.607
**MtDNA, mean (SD), log**_**10**_ **copies/mL**				
*ND2*	6.9 ± 0.5	7.0 ± 0.4	6.8 ± 0.5	0.212
*D-loop*	6.5 ± 0.7	6.5 ± 0.7	6.5 ± 0.7	0.927
**NDNA, mean (SD), log10 copies/mL**				
*GAPDH*	4.2 ± 0.5	4.2 ± 0.4	4.1 ± 0.6	0.483
*β-globin*	3.8 ± 0.5	3.8 ± 0.5	3.7 ± 0.6	0.746
**Urinary DAMPs levels, mean (SD), log**_**10**_ **copies/mL**				
*ND2*	7.9 ± 0.5	7.9 ± 0.5	7.8 ± 0.6	0.663
*D-loop*	7.8 ± 0.8	7.9 ± 0.5	7.4 ± 1.4	0.384
*GAPDH*	4.5 ± 0.9	4.6 ± 1.0	4.2 ± 0.8	0.389
*β-globin*	4.5 ± 1.0	4.6 ± 1.1	4.1 ± 0.9	0.262
**OUTCOMES**				
Hospital LOS, median (IQR), d	24.0 [13.5–41.0]	24.0 [13.8–46.5]	24.0 [12.0–32.0]	0.321
ICU LOS, median (IQR), d	15.0 [10.0–25.5]	20.5 [11.5–27.8]	12.0 [7.0–11.0]	0.418
Duration of mechanical ventilation, median (IQR), d	9.0 [0.0–23.5]	12.5 [2.5–32.0]	0.0 [0.0–9.0]	**0.005**
Duration of non-CVVH, median (IQR), d	16.0 [5.5–25.5]	16.0 [4.0–28.3]	8.0 [17.0–23.0]	**0.006**
Duration of (any) vasopressor, median (IQR), d	1.0 [0.0–8.0]	2.0 [0.0–10.5]	0.0 [0.0–5.0]	**0.000**
Hospital mortality, *n* (%)	25 (58.1)	22 (66.7)	3 (30.0)	**0.046**
28-day mortality, *n* (%)	16 (37.2)	14 (42.4)	2 (20.0)	0.233
**SITE OF INFECTION**, ***n*** **(%)**				
Lung		14 (42.4)		
Abdomen		15 (45.5)		
Catheter		3 (9.1)		
Unknown		1 (3.0)	

*BMI, body mass index; APACHE, Acute Physiology and Chronic Health Evaluation; SOFA, Sepsis-related Organ Failure; CVVH, continuous veno–venous hemofiltration; BUN, blood urea nitrogen; eGFR, estimated glomerular filtration; NGAL, neutrophil gelatinase-associated lipocalin; ALT, Alanine aminotransferase; AST, Aspartate amino transferase; CRP, C-reaction protein; PCT, procalcitonin; RBC, red blood cell; PLT, platelet; APTT, activated partial thromboplastin time; PT, prothrombin time; DAMPs, Damage-Associated Molecular Patterns; mtDNA, mitochondrial DNA; nDNA, nuclear DNA; HSP70, Heat Shock Protein 70; HMGB1, high-mobility group box 1 protein; INR, International Normalized Ratio; LOS, length of stay; ICU, Intensive care unit; IQR, interquartile range; SD, standard deviation*.

*Normally distributed data are presented as the mean (SD) (analysis of variance); non-normally distributed data are presented as median (IQR) (nonparametric Mann–Whitney U-tests); and categorical variables are presented as n (%) (chi-square test). Data in bold reflected P values < 0.05*.

AKI patients with sepsis were significantly older compared with those without sepsis (48.9 ± 13.9 vs. 37.3 ± 9.4 years, respectively; *P* = 0.042) and had markedly lower 24-h urinary output (280 vs. 750 mL, respectively; *P* = 0.009) at the time of enrollment. At baseline, clinical indices [i.e., Acute Physiology and Chronic Health Evaluation (APACHE) II score, Sequential Organ Failure Assessment (SOFA) score, cytokine levels, and DAMPs levels] were comparable between the two groups.

However, the outcomes were distinct between the two groups. The median duration of CVVH for the treatment of AKI patients with sepsis was longer than that for non-sepsis patients (8 vs. 5 days, respectively; *P* = 0.042). The median duration of mechanical ventilation was significantly longer in the AKI with sepsis group (12.5 vs. 0 days, respectively; *P* = 0.005). Moreover, in-hospital mortality was significantly higher in the AKI with sepsis group vs. the AKI without sepsis group (66.7 vs. 30.0%, respectively; *P* = 0.042) (Table 1). Of note, CVVH parameters were similar between the two groups, except for treatment duration.

### Clinical Indicators and Cytokine Levels During CVVH

We classified the AKI patients with sepsis into two groups, namely “survivors” and “non-survivors” to evaluate clinical data trends (Figure S1). As expected, most of the indices of liver and kidney function (e.g., levels of liver enzymes, serum creatinine, and blood urea) were improved during the first 7 days after initiation of CVVH, irrespectively of patient survival. However, in non-survivors, the level of total bilirubin temporarily returned within the normal range but rapidly increased after termination of CVVH. In contrast, the urine output did not recover during CVVH.

**Figure 2:**
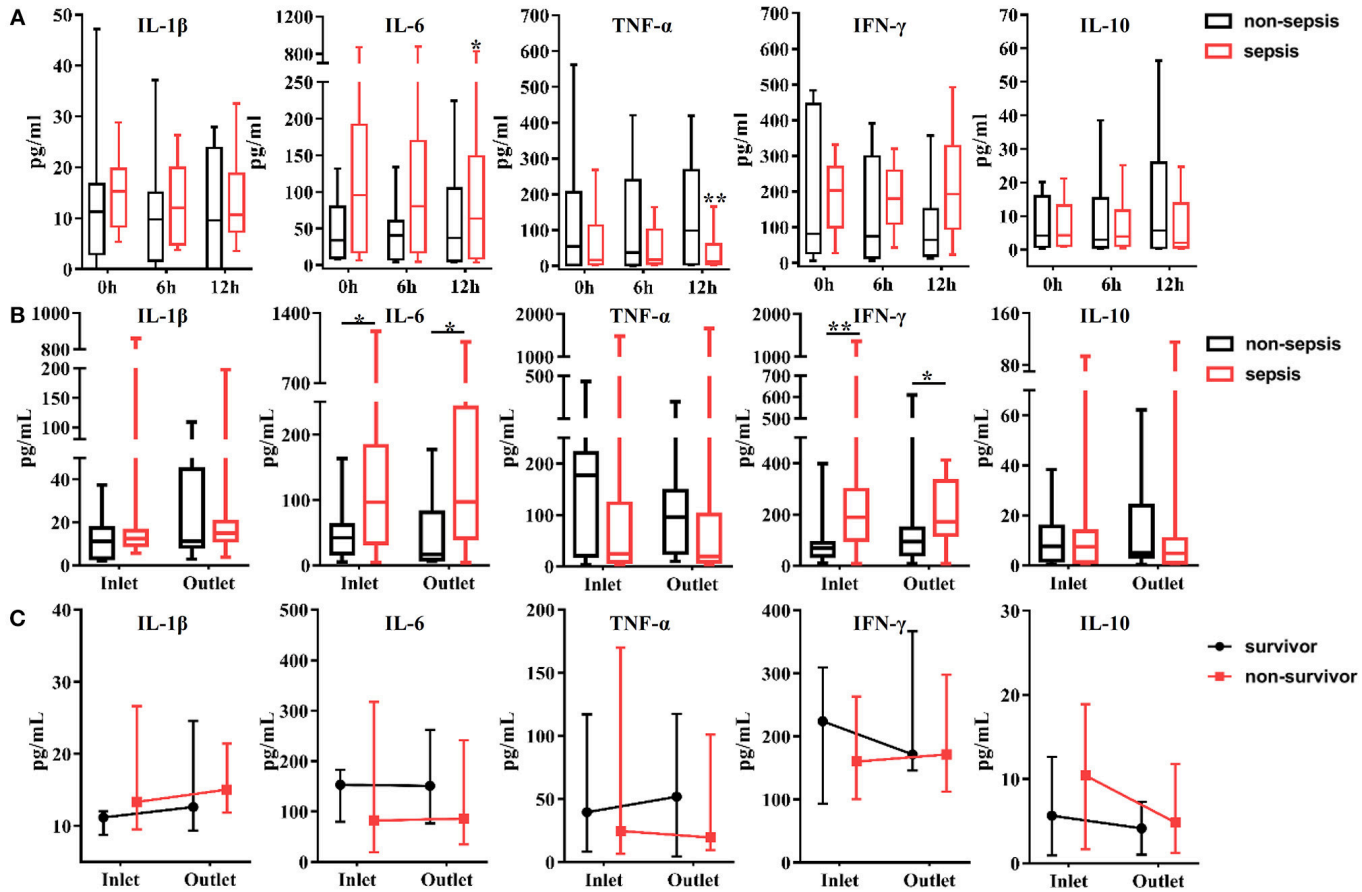
Effects of continuous veno-venous hemofiltration (CVVH) on levels of circulating cytokines. Cytokines, including IL-1b, IL-6, IFN-γ, TNF-α, and IL-10 were measured at baseline, 6, 12 h of CVVH from inlet and outlet of the filter. **(A)** Tendency of cytokines levels during the first 12 h was analyzed by repeated measure ANOVA in sepsis and non-sepsis groups, respectively. **(B)** Serum levels of cytokines at inlet and outlet in sepsis and non-sepsis groups (Median, IQR). **(C)** Comparisons of serum levels of cytokines between inlet and outlet in survived or non-survived septic patients. Data are presented as median and interquartile range. Error bars of the line chart denote the median with IQR. *P*-values indicating differences between the patients of two groups were calculated using Mann–Whitney *U*-tests. ^*^P < 0.05, ^**^P < 0.01. IFN, interferon; IL, interleukin; TNF, tumor necrosis factor; CVVH, continuous veno-venous hemofiltration; IQR, interquartile range.

The levels of cytokines were measured from the inlet and outlet of the CVVH dialyzer at baseline, 6, and 12 h after initiation of CVVH to evaluate the effect of CVVH on cytokine removal. Only the levels of IL-6 and TNF-α were decreased in the AKI with sepsis group (*P* = 0.046 and *P* = 0.008, respectively) (Figure 2A). There was no difference in the levels of all measured cytokines between the inlet and outlet, regardless of the presence or absence of sepsis in AKI patients (Figure 2B). Furthermore, the levels of circulating cytokines were similar between the surviving and non-surviving AKI patients with sepsis (Figure 2C).

### Circulating and Urinary Levels of DAMPs During CVVH

**Figure 3:**
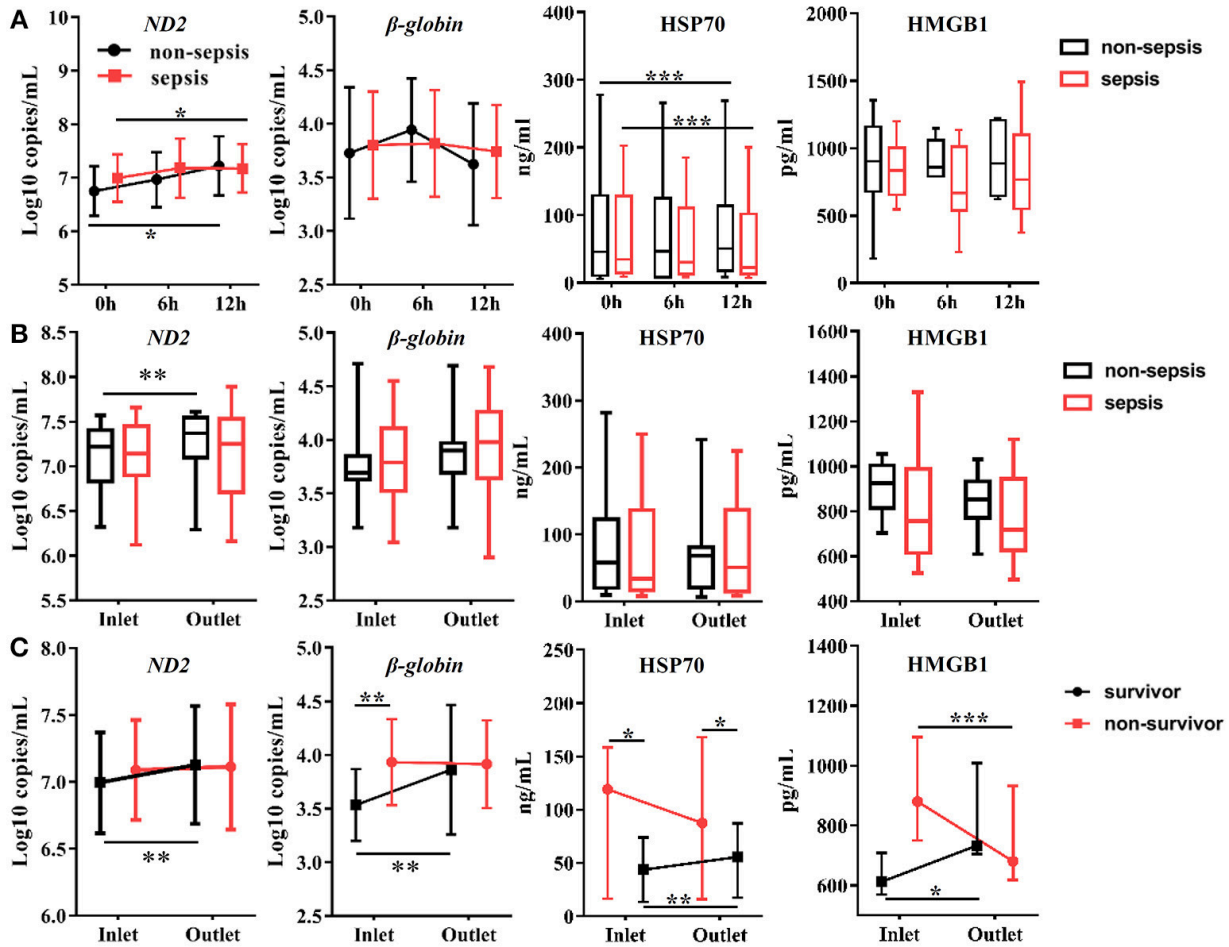
Effects of CVVH on levels of circulating Damage-Associated Molecular Patterns (DAMPs). DAMPs, including mitochondrial DNA (*ND2*), nuclear DNA (β*-globin*), HSP70, and HMGB1, were measured at baseline, 6, 12 h of CVVH from inlet and outlet of filter. **(A)** Tendency of DAMPs levels during the first 12 h was analyzed by repeated measure ANOVA in sepsis and non-sepsis groups, respectively. Error bars of the line chart denote the mean with SD. **(B)** Box plots shown the levels of DAMPs at inlet and outlet in sepsis and non-sepsis groups. **(C)** Comparisons of mean levels of mtDNA and nDNA, and levels of HSP70 and HMGB1 (median ± IQR) between inlet and outlet in survived or non-survived septic patients. Comparison of continuous variables between the two groups was conducted with the Student's *t*-test or Mann–Whitney *U*-test depending on Gaussian distribution. ^*^P < 0.05, ^**^P < 0.01, ^***^P < 0.001. DAMPs, damage-associated molecular patterns; mtDNA, mitochondrial DNA; nDNA, nuclear DNA; HSP70, heat shock protein 70; HMGB1, high-mobility group box 1 protein; CVVH, continuous veno-venous hemofiltration.

The circulating levels of DAMPs, including mtDNA (*ND2, D-loop*), nDNA [*glyceraldehyde 3-phosphate dehydrogenase (GAPDH)*, β*-globin]*, HSP70, and HMGB1, were measured at the same time points as those used for the measurement of cytokine levels. All plasma samples exhibited detectable levels of DAMPs. In the first 12 h after initiation of CVVH, the levels of mtDNA were increased in all AKI patients, regardless of the presence or absence of sepsis (*ND2*: *P* = 0.035; *D-loop*: *P* = 0.003; Figure 3A, Figure S2). The level of HSP70 in the plasma was reduced in both AKI patients with sepsis (*P* = 0.000) and without sepsis (*P* = 0.001). However, this trend was particularly apparent in the former group. The levels of nDNA in the plasma, including *GAPDH*, β*-globin*, and HMGB1 were unaltered during the first 12 h (Figure 3A, Figure S2). In brief, the tendency of change in the levels of DAMPs during CVVH was similar between the sepsis and non-sepsis group.

In addition, we compared the levels of DAMPs between the inlet and outlet of the dialyzer at the same time points to determine the effect of CVVH on the rates of DAMP clearance. We did not find differences between the inlet and outlet data in the sepsis or non-sepsis group, except an increase in the level of *ND2* after blood passage through the filter in non-sepsis group (inlet: 7.08 ± 0.40 log_10_ copies/mL; outlet: 7.24 ± 0.41 log_10_ copies/mL; *P* = 0.008) (Figure 3B). We further divided the patients with sepsis into a survivor and non-survivor group. The circulating concentrations of DAMPs, including mtDNA, nDNA, HSP70, and HMGB1, were increased in the survivor group after blood passage through the dialyzer (Figure 3C, Figure S2). Notably, the levels of HMGB1 were decreased significantly in the non-survivor group (Figure 3C).

**Figure 4:**
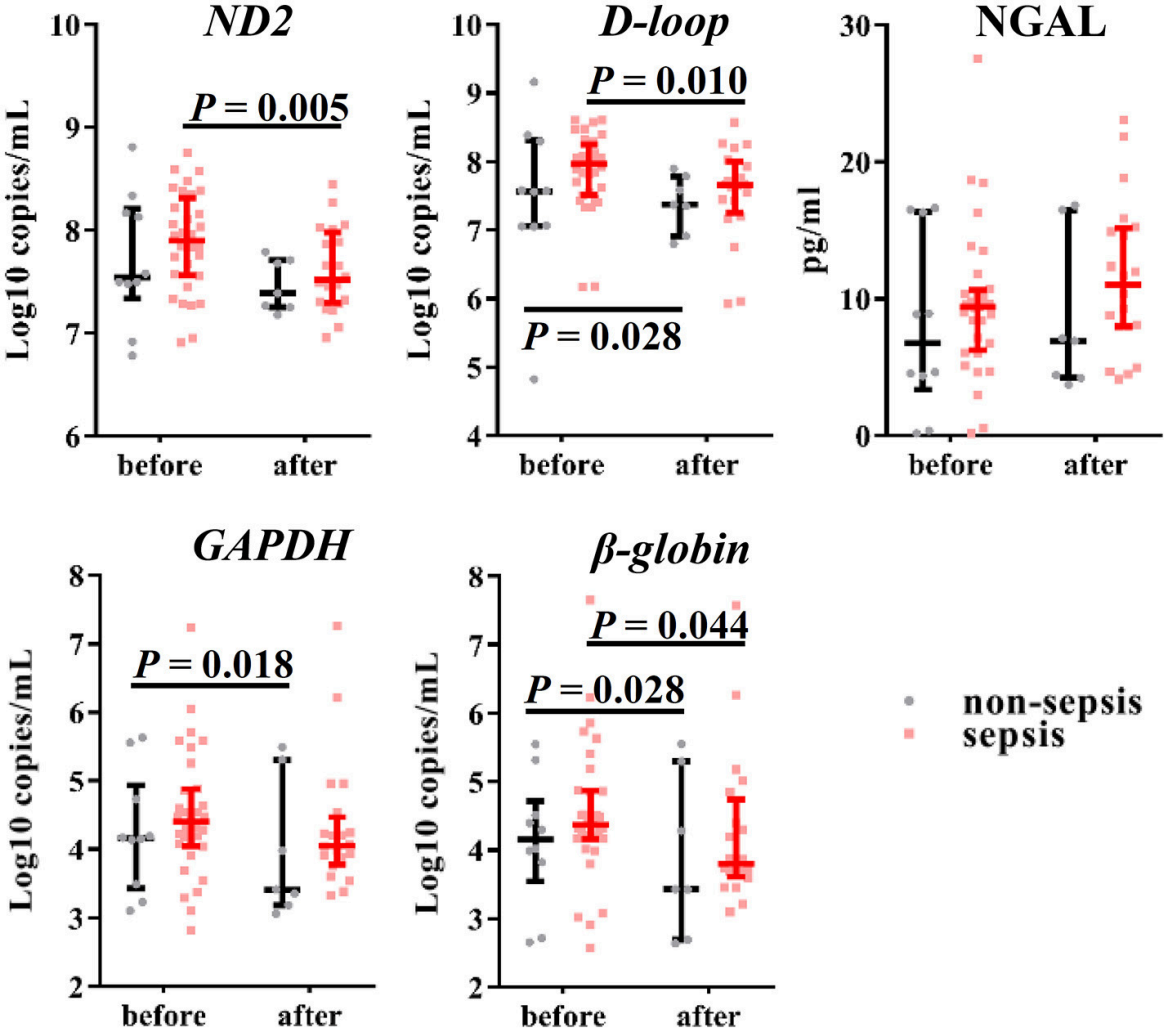
Effects of CVVH on levels of urinary mtDNA, nDNA and NGAL. Mitochondrial DNA (*ND2, D-loop*), nuclear DNA (*GAPDH*, β*-globin*) and NGAL in urine were measured at baseline and end of CVVH. Error bars denote the median and interquartile range. *P*-values indicating differences between the patients of two groups were calculated using Mann–Whitney *U*-tests. mtDNA, mitochondrial DNA; nDNA nuclear DNA; NGAL, neutrophil gelatinase-associated lipocalin; CVVH, continuous veno-venous hemofiltration.

In our previous study, we demonstrated the effectiveness of using the urinary levels of mtDNA as evidence of renal mitochondrial injury induced by AKI after sepsis. Thus, we measured the urinary levels of NGAL and cf-DNA at baseline and end of CVVH to evaluate renal function. The urinary levels of nDNA and mtDNA were reduced after CVVH (Figure 4), unlike that of NGAL (despite an observed increasing trend, *P* = 0.257), regardless of the presence or absence of sepsis in AKI patients. Consistently, clinical indicators for the evaluation of renal function (i.e., serum creatinine, blood urea nitrogen, and estimated glomerular filtration) were also reduced following treatment with CVVH (Figure S1). Therefore, CVVH may (at least partly) improve renal function.

### Relationship Between the Clearance Rate of DAMPs, Level of mHLA-DR, and Outcomes

To further investigate the effect of DAMP removal in AKI patients with sepsis, we calculated their clearance rate and assessed its association with the outcome and immune state. The clearance rate is similar to the meaning of the creatinine clearance rate in the kidney, which represents the net result of the production and clearance rates of the molecules. Thus, its value depends on the integrated effects of production by the cells and clearance by the filter. Higher values indicate high clearance rates vs. production rates.

**Figure 5:**
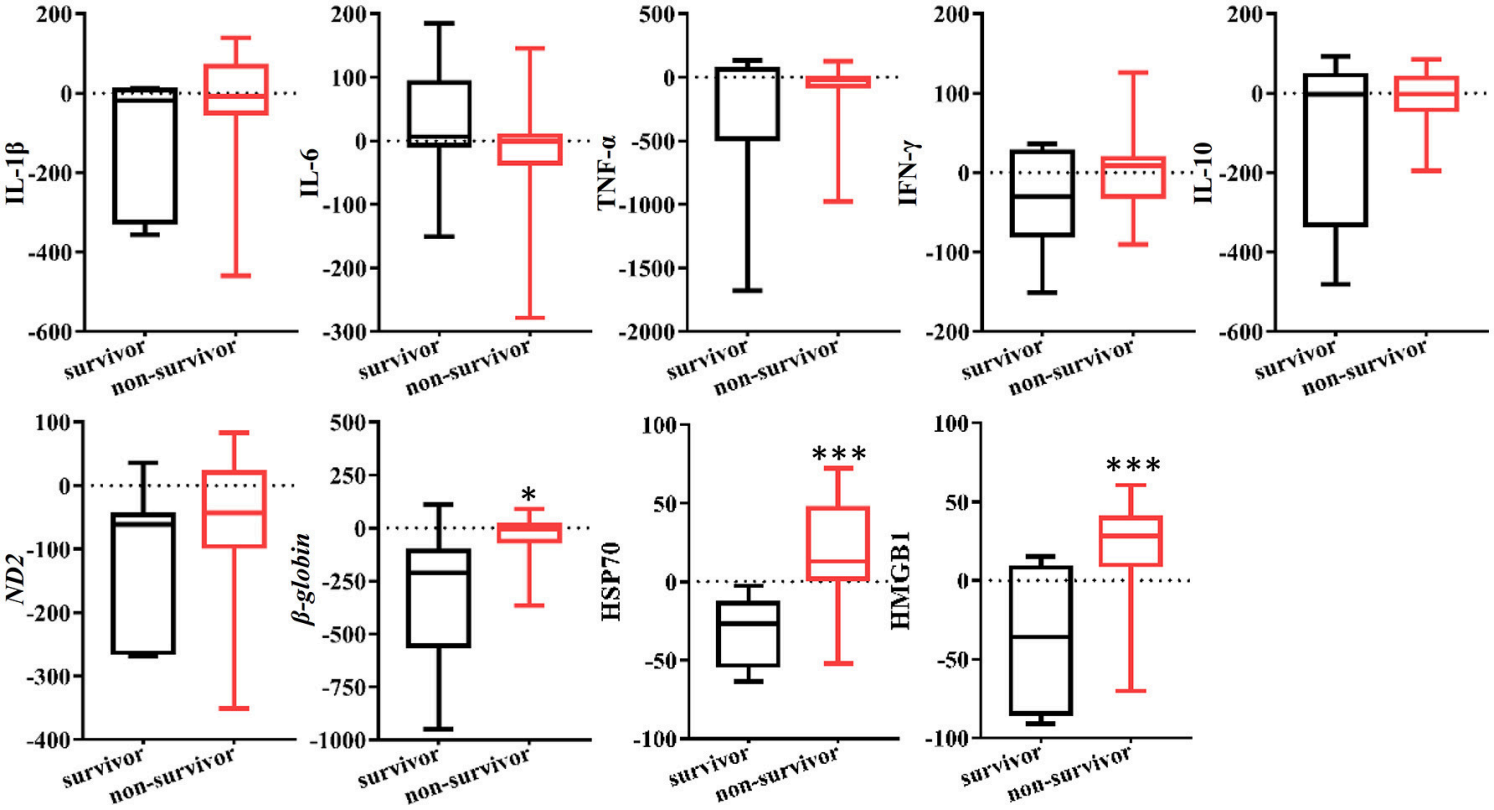
Clearance rate of cytokines and DAMPs in survived or non-survived septic patients. Box plots showed the levels of cytokines and DAMPs clearance rate AKI patients with sepsis. *P*-values indicating differences between the patients of two groups were calculated using Mann–Whitney *U*-tests. ^*^P < 0.05, ^***^P < 0.001. IFN, interferon; IL, interleukin; TNF, tumor necrosis factor; HSP70, heat shock protein 70; HMGB1, high-mobility group box 1 protein.

**Figure 6:**
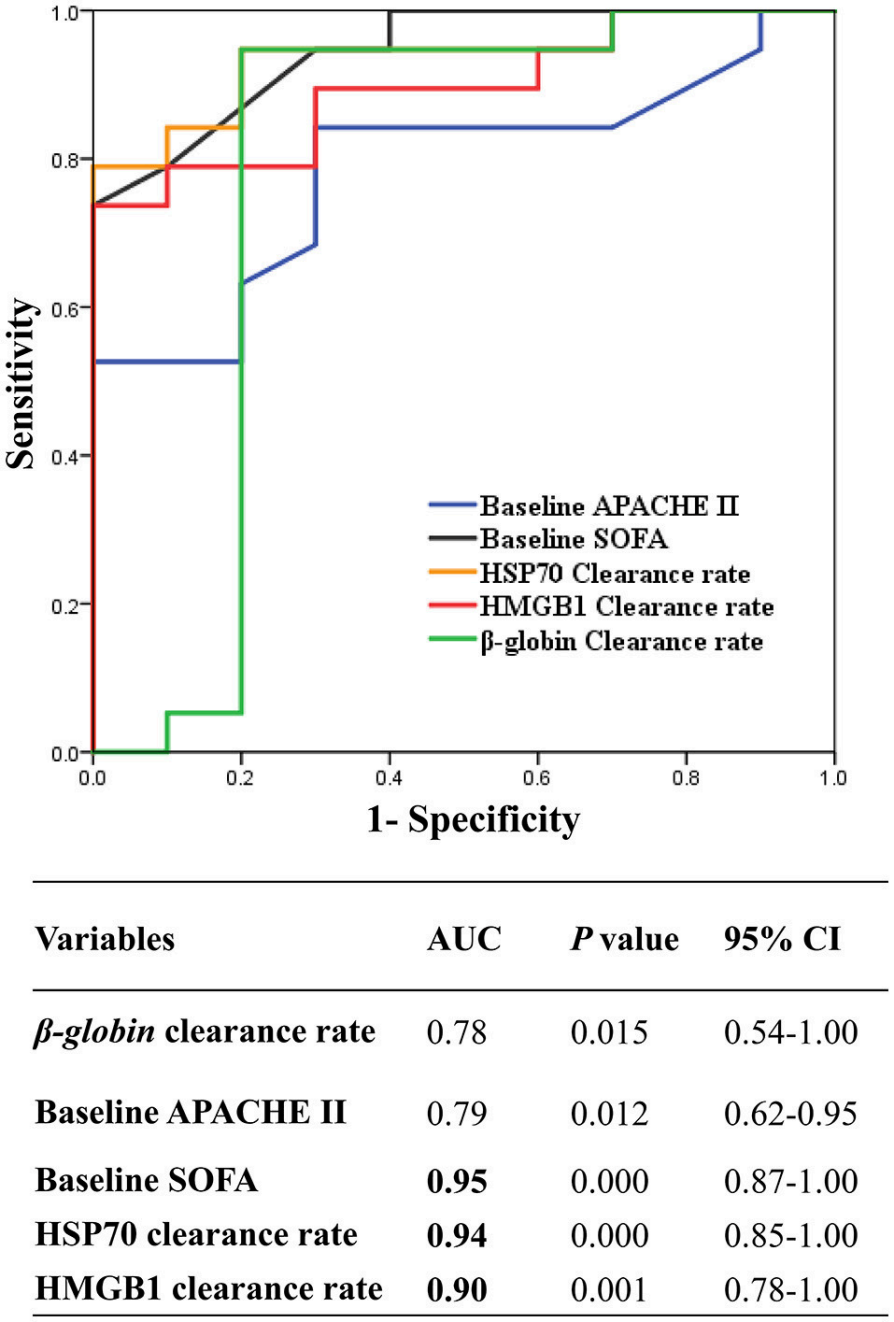
Receiver Operating Characteristic curves (ROC) of the HSP70, HMGB1, and β*-globin* clearance to predict hospital mortality. ROC curve of the mean of β*-globin* clearance rate and HMGB1 clearance rate, and HSP70 clearance rate, and baseline APCHE II, SOFA score. AUC, area under curve; CI, confidence interval; APACHE, Acute Physiology and Chronic Health Evaluation; SOFA, Sepsis-related Organ Failure; CVVH, continuous veno-venous hemofiltration; HSP70, heat shock protein 70; HMGB1, high-mobility group box 1 protein.

**Figure 7:**
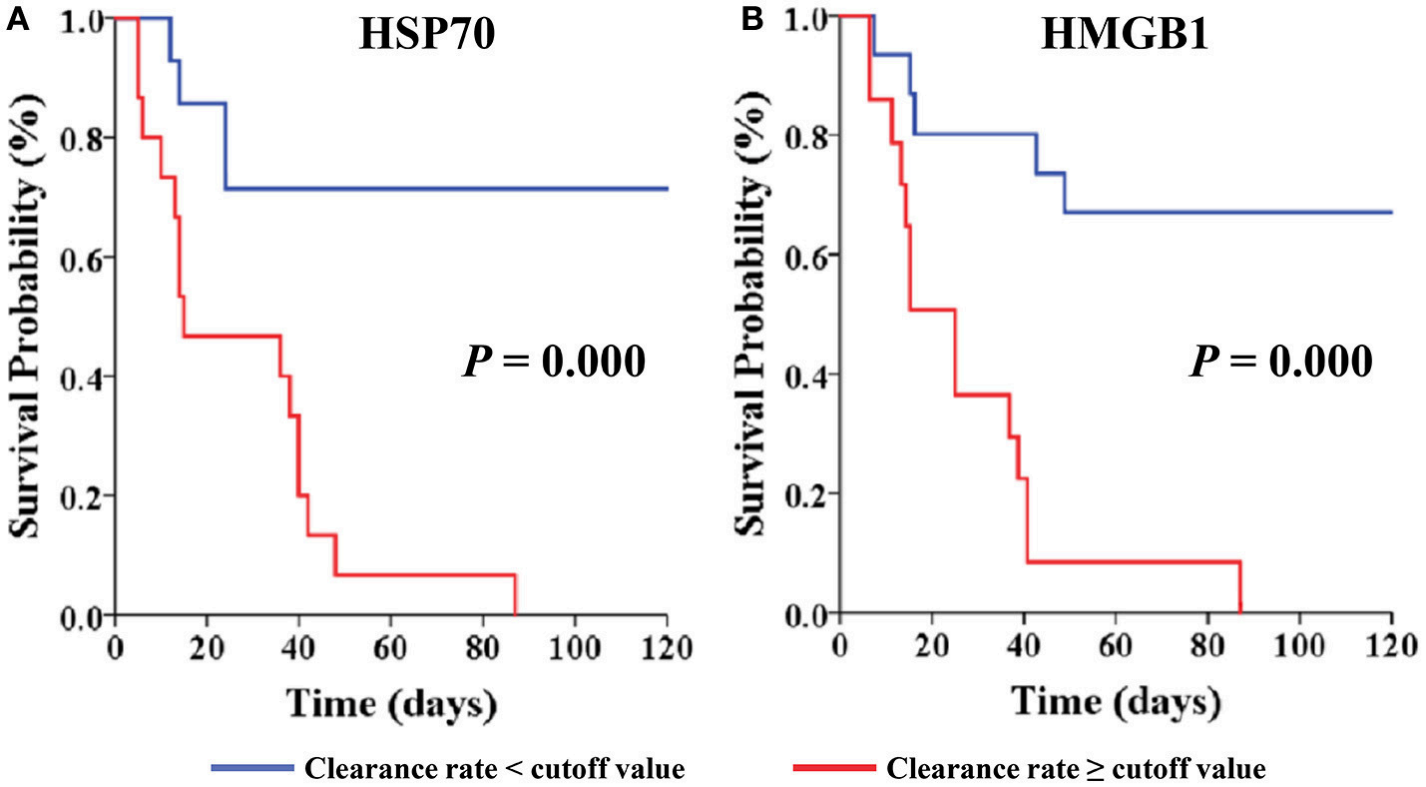
Kaplan-Meier Survival by cut-off value of clearance rate for the AKI with septic patients. Patients are stratified by cut-off value of HSP70 clearance rate **(A)** and HMGB1 clearance rate **(B)**. Patients were censored from survival analysis after discharge. HSP70, heat shock protein 70; HMGB1, high-mobility group box 1 protein.

In the present study, the clearance rates of β*-globin*, HSP70, and HMGB1 were higher in all non-surviving AKI patients with sepsis (*P* = 0.006, *P* = 0.005, and *P* = 0.000, respectively) (Figure 5). Furthermore, we performed analyses of ROC curves to determine predictors of mortality (Table S1), including the circulating levels of DAMPs as well as the clearance rates of cytokines and DAMPs. The clearance rates of HSP70, HMGB1, and β*-globin* were good predictors of mortality (Figure 6). Analyses of the area under the curve (AUC) of ROC curves showed that the clearance rate of β*-globin* was similar to that of the APACHE II score at baseline. Notably, the clearance rates of HSP70 and HMGB1 exhibited a similar prediction efficiency to that of the SOFA score at baseline. Consistent with these findings, the Kaplan–Meier survival analysis revealed that patients with higher HSP70 or HMGB1 clearance rate were associated with significantly higher risk of mortality than those with lower clearance rate (log-rank test: *P* = 0.000 for both) (Figure 7).

**Figure 8:**
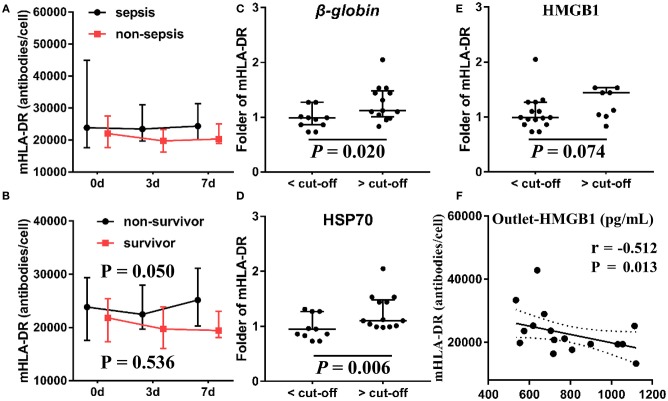
Association between mHLA-DR, outcome and DAMPs clearance. The levels of mHLA-DR were inspected at baseline, 3, 7 day after the CVVH initiation. Tendency of mHLA-DR changes (median ± IQR) was analyzed by repeated measure ANOVA in sepsis and non-sepsis groups **(A)** or survived and non-survived septic patients **(B)** The subjects were divided into two groups based on the cut-off value of the clearance rates of β*-globin* **(C)**, HSP70 **(D)**, and HMGB1 **(E)** to compare the difference of mHLA-DR fold change on day 7 after CVVH initiation, *P*-values indicating differences between the patients of two groups were calculated using Mann–Whitney *U*-tests. **(F)** Correlations between the levels of mHLA-DR on 7 day and the level of HMGB1 at outlet were determined using Spearman correlation test. CVVH, continuous veno-venous hemofiltration; DAMPs, damage-associated molecular patterns; mHLA-DR, mononuclear human leukocyte antigen-DR; HSP70, heat shock protein 70; HMGB1, high-mobility group box 1 protein.

In addition, we measured the level of mHLA-DR—an indicator of the immune state—at 0, 3, and 7 days after initiation of CVVH to evaluate the immune state of the patients. We found no difference in the level of mHLA-DR expression between AKI patients with sepsis and those without sepsis (median ± IQR) (Figure 8A). However, the level of mHLA-DR was increased in AKI patients with sepsis who expired (*P* = 0.05) (Figure 8B). To ascertain the relationship between the clearance rate of DAMPs and the change in the level of mHLA-DR in AKI patients with sepsis, we classified patients into two groups based on the cut-off value of DAMPs (Figures 8C–E). Interestingly, the level of mHLA-DR was significantly increased in patients with higher β*-globin* and HSP70 clearance rates than the cut-off value (*P* = 0.02 and *P* = 0.006, respectively). Similarly, there was a tendency toward increase in the level of mHLA-DF in patients with higher HMGB1 clearance rate than the cut-off value (*P* = 0.074). In addition, the level of HMGB1 at the outlet was negatively related with the level of mHLA-DR (Spearman rank correlation coefficient = −0.512, *P* = 0.013) (Figure 8F).

### Multivariate Logistic Regression Analysis

We performed univariate and multivariate logistic regression analyses to examine other baseline or disease-related factors and evaluate the contribution of the clearance rates of β*-globin*, HSP70, or HMGB1 to mortality. These analyses revealed that only the clearance rate of HSP70 remained independently associated with high mortality after adjustment for age, APACHE II score, SOFA score, urine output, level of total bilirubin, and coagulation indicators [odds ratio (OR): 1.025; 95% confidence interval (CI): 1.012–1.039; *P* = 0.000] (Table 2).

**Table 2:** Mortality prediction and HSP70 removed amount rate on CVVH.

	**Logistic regression**		
	***P-*value**	**Odds ratio**	**95% CI**
Clearance of HSP70 unadjusted	0.000	1.067	1.032–1.103
Adjusted for age and APACHE II score	0.001	1.068	1.027–1.110
Adjusted for age, APACHE II score, SOFA score, 24h urine output, eGFR and PLT count	0.000	1.028	1.014–1.042
Adjusted for age, APACHE II score, SOFA score, 24h urine output, CRP, eGFR, PLT count, APTT and total bilirubin	0.000	1.025	1.012–1.039

*APACHE, Acute Physiology and Chronic Health Evaluation; SOFA, Sepsis-related Organ Failure; eGFR, estimated glomerular filtration; CRP C, reaction protein; PLT, platelet; APTT, activated partial thromboplastin time*.

In addition, we analyzed the contribution of the urinary levels of DAMPs to the duration of CVVH. The urinary level of β*-globin* was an independent factor for the duration of CVVH even after adjustment for age, APACHE II score, SOFA score, urine output, level of creatinine in the serum, estimated glomerular filtration rate, and level of blood urea (standardization regression coefficient: 0.460; 95% CI: 1.720–8.857, *P* = 0.005) (Table 3).

**Table 3:** Prediction for duration of CVVH.

	**Linear regression**		
	***P-*value**	**sRE**	**95% CI**
Urinary *β-globin* level before the CVVH initiation unadjusted	0.005	0.481	1.774–9.291
Adjusted for age and APACHE II score	0.005	0.481	1.774–9.421
Adjusted for age, APACHE II score, SOFA score, 24h urine output	0.005	0.460	1.720–8.857
Adjusted for age, APACHE II score, SOFA score, 24h urine output, serum creatinine, eGFR and blood urea	0.005	0.460	1.720–8.857

*sRE, standardization regression coefficient; CVVH, continuous veno–venous hemofiltration; APACHE, Acute Physiology and Chronic Health Evaluation; SOFA, Sepsis-related Organ Failure; eGFR, estimated glomerular filtration*.

## Discussion

This was the first study to evaluate the efficiency of CVVH for the removal of DAMPs and the effects of this removal. Our study demonstrated three key findings. Firstly, the newly identified urinary indicators of renal injury (i.e., mtDNA and nDNA) recovered after CVVH and the level of urinary nDNA was an independent prognostic factor for the duration of CVVH. Secondly, the efficiency of CVVH for the removal of cytokines and DAMPs was variable. The levels of IL-6, TNF-α, and HSP70 decreased within the first 12 h of CVVH in AKI patients with sepsis, whereas the levels of DAMPs at the outlet were temporarily increased after blood passage from the inlet through the dialyzer in survivor AKI patients with sepsis. Finally, the higher clearance rate of DAMPs (especially HSP70) was significantly associated with poor outcomes and immune disorders.

CRRT is undoubtedly beneficial for patients with lethal electrolyte abnormalities, renal dysfunction, or liver dysfunction (15). In our previous study, we demonstrated that the urinary levels of nDNA and mtDNA are novel biomarkers of AKI, and may be used as evidence of renal mitochondrial injury induced by AKI after sepsis (10). Our results revealed that the urinary levels of nDNA and mtDNA decreased after CVVH in all patients, and urinary nDNA (β*-globin*) was an independent prognostic factor for the duration of CVVH. Furthermore, we observed improvement in the indices of renal and liver function, especially the levels of creatinine in the serum, blood urea, and liver enzymes. Therefore, we infer that CVVH may (at least partly) improve kidney injury in terms of histopathology. Nevertheless, the 24-h urinary volume did not recover during CVVH in patients who eventually expired. Perez-Fernandez et al. also emphasized that a low urine output was associated with poor outcome in AKI patients with sepsis (22). Considering that the urine output was also dependent on the circulatory function, persistently low urine output may be mainly due to disease progression-induced disturbances in microcirculation.

Our previous research revealed that the concentration of cf-DNA increased in patients with severe disease (20). Accordingly, we found that the level of nDNA correlated with prognosis. In addition, in surviving AKI patients with sepsis, the concentrations of DAMPs were markedly increased after blood passage through the inlet of the dialyzer, but declined or recovered prior to the subsequent sampling time point. DAMPs were detected in the filtrate (data not shown), suggesting that clearance through CVVH was reliable. However, in non-survivors, the level of DAMPs remained high. These results suggest defects in the elimination of DAMPs in non-survivors.

Although studies reported that the levels of IL-6 and TNF-α decreased with time (6), there is currently no consensus to guide clinicians in terms of controlling the levels of inflammation and DAMPs in AKI patients with sepsis managed through CRRT. This may be attributed to a lack of evidence and deep insight into the effects of CRRT on the human body (23).

In this study, there was no correlation between cytokine clearance and mortality. Chung et al. also showed that high-volume hemofiltration did not reduce mortality compared with standard treatment, despite the extensive removal of pro-inflammatory cytokines in patients treated with high-volume hemofiltration (17). Park et al. also performed a randomized controlled trial, demonstrating that a high dose of CVVH (80 mL/kg/h) did not improve outcomes in sepsis-associated AKI patients despite its considerable effect on the removal of pro-inflammatory cytokines (16). Thus, CRRT-induced changes in the levels of cytokines may not be sufficient to influence clinical endpoints. Moreover, in recent years, a new concept termed “dialysis trauma” suggests that dialysis involves microcirculation (24). Our findings suggest that elimination of DAMPs worsened the outcome in AKI patients with sepsis undergoing CVVH. The net outcome is dependent on a balance of the detrimental effects vs. the protective effects of CVVH. This may explain the inadequacy of CRRT-induced changes in the levels of pro-inflammatory factors to influence clinical outcomes.

Several studies have investigated the roles of DAMPs in critical-care illness and sepsis (6, 25, 26). However, there are no studies addressing the clearance rate of DAMPs in CVVH and its effects on the outcomes and immune homeostasis. In this study, data revealed that there was no difference between patients with or without sepsis. Thus, we excluded the effect of the disease on DAMPs and cytokines during the first 12 h of CVVH. Furthermore, we compared the difference between the non-survivors and survivors in the sepsis group. A higher level of mHLA-DR was linked to higher mortality. In our study, AKI patients with sepsis who expired had markedly higher levels of mHLA-DR than patients in other studies (27, 28). The mHLA-DR is currently the “gold standard” for the identification of immunosuppression and participates in antigen presentation and perpetuation of the inflammatory reaction (29, 30). Hence, a higher level of mHLA-DR may reflect excessive immune activation. Accordingly, we demonstrated that the clearance rates of HSP70 and HMGB1 were higher in AKI patients with sepsis who expired. The clearance rates of HSP70 and HMGB1 increase in parallel with the level of mHLA-DR, indicating that the levels of DAMPs may play an anti-inflammatory role. Consistently, several studies have demonstrated that DAMPs may induce immune suppression and regulate immune response. This has been specifically described for HMGB1 (31, 32) and HSP70 (33, 34). Scholars have demonstrated that the extracellular levels of HSP70 activate and suppress the immune response *via* the paired receptors sialic acid-binding immunoglobulin-like lectins Siglec-5 and Siglec-14 (34–36). Thus, excessive removal of HSP70 during CVVH may amplify the inflammatory process, disrupt immune homeostasis, and exacerbate disease.

Timmermans et al. reported that, in trauma patients, released DAMPs are associated with an acute, predominantly anti-inflammatory response, and a suppressed state of the immune system (28). Schafer et al. also demonstrated that mtDNA may be a link between initial inflammation and subsequent immunosuppression in critically ill patients, probably through a Toll-like receptor-9 pathway (37). Leijte et al. (38) have shown that increased levels of plasma DAMPs were associated with immune suppression and post-operative infections in patients undergoing cytoreductive surgery and hyperthermic intraperitoneal chemotherapy. Our results showed that extensive clearance of DAMPs was associated with an extremely high level of mHLA-DR expression and higher mortality. Collectively, these results imply that DAMPs, especially HSP70 and HMGB1, may act as anti-inflammatory molecules involved in the modulation of the immune system. Excessive removal of these molecules during CVVH may exacerbate disease and amplify the inflammatory process. Consequently, patients may expire due to an exaggerated inflammatory response at the early stage of sepsis.

This study had several shortcomings. Firstly, the patient population was small. Further, studies with larger samples are warranted to confirm the present results. Although the sample size of this study was small, our novel findings are important for the management of AKI patients with sepsis in the clinical setting. Secondly, we did not compare the different modes of RRT (e.g., high dose vs. conventional dose) or the types of filtration membrane. Hence, we were unable to reach a conclusion regarding the preferred type of membrane or mode of CRRT. Nevertheless, the process of CVVH was consistent throughout the study period, avoiding potential confounders. In addition, we did not investigate sepsis patients who were not treated with CVVH. It is not possible to calculate the clearance rate in a non-CVVH group. Moreover, the pathophysiological mechanisms of sepsis in the absence of CVVH are different from those involved in the population of the present study, regardless of disease severity. Consequently, an investigation of differences between the outcomes of sepsis patients with or without CVVH would be meaningless. Thirdly, there were confounders (e.g., early initiation of antibiotic therapy, source control, and vasopressor support) in our study during CVVH. However, we collected the samples during the first 12 h to avoid the influence of other long-term treatments on the clearance rate. Continuous monitoring of clinical data showed that disease severity was similar during the first day of CVVH. In addition, several studies have suggested that the nDNA level is linked to disease severity (39) and our data showed that the circulating levels of nDNA during the first 12 h of CVVH were almost constant. Thus, these clinical confounders did not influence the conclusions of this study. Fourthly, there was a lack of data regarding the long-term impact of changes in the levels of DAMPs on the levels of mHLA-DR and prognosis.

Despite the aforementioned limitations, our study has important implications for clinicians. Mortality among patients in the intensive care unit remains high, despite the latest advances and innovations in CRRT (17). Most of the researchers and physicians are focused on the role of CRRT in reducing the levels of cytokines. However, despite the extensive removal of pro-inflammatory cytokines in the high-volume hemofiltration (HVHF) groups, CRRT did not reduce mortality compared with standard treatment (16). Our results indicated that extensive removal of DAMPs during CVVH was associated with poor prognosis, which may neutralize the beneficial effect of this treatment. Therefore, it is imperative to develop new filtration membranes–with improved biocompatibility to reduce the stress of circulatory cells–and individualized hemofiltration strategies by monitoring the change in the levels of DAMPs. Collectively, we do not recommend the use of CVVH merely for the removal of pro-inflammatory factors in the treatment of sepsis due to the complex effects of CVVH on DAMPs. In addition, extensive elimination of DAMPs may intensify the immune imbalance in patients with sepsis. This hypothesis is consistent with the recommendations included in the guidelines of the 2016 Surviving Sepsis Campaign (23). An advanced understanding of the host response during CVVH is necessary to optimize hemofiltration, shorten the course of AKI, reduce injury to distant organs, and improve survival.

## Conclusions

The urinary levels of mtDNA and nDNA recovered after CVVH, and the level of nDNA was an independent prognostic factor for the duration of CVVH. These findings indicated that CVVH can alleviate kidney injury. However, the efficiency of CVVH for the removal of cytokines and DAMPs was variable and complex. The circulating levels of DAMPs were rapidly and temporarily increased after blood–obtained from surviving AKI patients with sepsis–passaging through the dialyzer. The levels of HSP70 and HMGB1 decreased in AKI patients with sepsis who expired. Moreover, the higher clearance rate of DAMPs, especially HSP70 and HMGB1, was significantly associated with immune disorders and poor prognosis. Thus, we do not recommend the use of CVVH in AKI patients with sepsis merely for the removal of inflammatory mediators without other definitive indications for CVVH.

## Data Availability

The datasets analyzed during the present study are available from the corresponding author on reasonable request.

## Author's Note

All authors are employees of the Department of Surgery, Jinling Hospital, 305 East Zhongshan Road, Nanjing, 210002, China. An abstract of these data has been accepted as an oral presentation at the 38th Annual Meeting of the Surgical Infection Society.

## Author Contributions

JW, JR, and XW conceived the study and interpreted the results. JR designed the study. JW, QL, QH, and ZH acquired the data. JW wrote the manuscript. All authors contributed to the study design and manuscript preparation, and read and approved the final manuscript.

### Conflict of Interest Statement

The authors declare that the research was conducted in the absence of any commercial or financial relationships that could be construed as a potential conflict of interest.

## Supplementary Material

Figure S1 Tendency of change in clinical data under CVVH in survived and non-survived septic AKI patients. Twenty-four clinical indexes, including blood routine, liver function, renal function, electrolyte concentration, and coagulation function, etc. were collected every day during the first 7 days after CVVH initiation to form the line chart. The values at “After” denotes the indexes were acquired within 1 day of termination of CVVH treatment. ^a^Tendency of change was significant in total patients (*P* < 0.05). ^#^Tendency was significant different between survived and non-survived sepsis patients (*P* < 0.05). ^Δ^The difference between before and after CVVH were significant distinct between survived and non-survived groups (*P* < 0.05). ^*^The values were significant different between survived and non-survived groups after finished CVVH treatment (*P* < 0.05). BUN, blood urea nitrogen; eGFR, estimated glomerular filtration; ALT, Alanine aminotransferase; AST, Aspartate amino transferase; CRP, C-reaction protein; PCT, procalcitonin; RBC, red blood cell; PLT, platelet; APTT, activated partial thromboplastin time; PT, prothrombin time; INR, International Normalized Ratio.

Figure S2 Effects of CVVH on *D-loop* (mtDNA) and *GAPDH* (nDNA) level and the clearance rate of them. Mitochondrial DNA (*D-loop*), nuclear DNA (*GAPDH*) were measured at baseline, 6, 12 h of CVVH at inlet and outlet. **(A)** Tendency of their levels during the first 12 h was analyzed by repeated measure ANOVA in sepsis and non-sepsis groups, respectively. Error bars of the line chart denote the mean with SD. **(B)** Box plots shown the levels of DAMPs at inlet and outlet in sepsis and non-sepsis groups. **(C)** Comparisons of mean levels of mtDNA and nDNA in survived or non-survived septic patients. **(D)** Box plots shown the levels of clearance rate (median ± IQR) in survived or non-survived septic patients. ^*^*P* < 0.05, ^**^*P* < 0.01, *P* < 0.001. DAMPs, damage-associated molecular patterns; mtDNA, mitochondrial DNA; nDNA, nuclear DNA; CVVH, continuous veno-venous hemofiltration; IQR, interquartile range.

Table S1 Receiver Operating Characteristic curves (ROC) of the clearance of DAMPs and cytokines to predict hospital mortality. Fold front denotes the significance of ROC analysis was <0.05. The cut-off values and corresponding sensitivity and specificity were calculated based on Youden index. IFN, interferon; IL, interleukin; TNF, tumor necrosis factor; DAMPs, Damage-Associated Molecular Patterns; mtDNA, mitochondrial DNA; nDNA, nuclear DNA; HSP70, Heat Shock Protein 70; HMGB1, high-mobility group box 1.

## Abbreviations

CVVH: Continuous veno-venous hemofiltration
DAMPs: Damage-Associated Molecular Patterns
mtDNA: mitochondrial DNA
nDNA: nuclear DNA
HSP70: Heat Shock Protein 70
HMGB1: High Mobility Group Box 1
cf-DNA: Cell-free DNA
TNF-α: tumor necrosis factor alpha
IFN-γ: interferon gamma
mHLA-DR: mononuclear human leukocyte antigen-DR
APACHE II: Acute Physiology and Chronic Health Evaluation II
SOFA: Sequential Organ Failure Assessment
ICU: Intensive care unit
NGAL: neutrophil gelatinase-associated lipocalin
ABC: antibodies bound per cell
IQR: interquartile range
KDIGO, Kidney Disease: Improving Global Outcomes
mCLcytokines: mean of cytokines clearance rate
mCLDAMPs: mean of DAMPs clearance rate
mCDAMPs: mean of circulating DAMPs levels.

## References

[1] Singer M Deutschman CS Seymour CW Shankar-Hari M Annane D Bauer M The third international consensus definitions for sepsis and septic shock (Sepsis-3). J Am Med Assoc. (2016) 315:801–10. 10.1001/jama.2016.0287 PMC4968574 26903338

[2] Bagshaw SM George C Bellomo R Committee ADM. Early acute kidney injury and sepsis: a multicentre evaluation. Crit Care (2008) 12:R47. 10.1186/cc6863 18402655 PMC2447598

[3] Kolhe NV Stevens PE Crowe AV Lipkin GW Harrison DA. Case mix, outcome and activity for patients with severe acute kidney injury during the first 24 hours after admission to an adult, general critical care unit: application of predictive models from a secondary analysis of the ICNARC Case Mix Programme database. Crit Care (2008) 12 (Suppl. 1):S2. 10.1186/cc7003 19105800 PMC2607110

[4] Quinto BM Iizuka IJ Monte JC Santos BF Pereira V Durao MS. TNF-alpha depuration is a predictor of mortality in critically ill patients under continuous veno-venous hemodiafiltration treatment. Cytokine (2015) 71:255–60. 10.1016/j.cyto.2014.10.024 25461406

[5] Marx D Metzger J Pejchinovski M Gail RB Frantzi M Latosinska A. Proteomics and metabolomics for AKI diagnosis. Semin Nephrol. (2018) 38:63–87. 10.1016/j.semnephrol.2017.09.007 29291763

[6] Simmons JD Lee YL Mulekar S Kuck JL Brevard SB Gonzalez RP. Elevated levels of plasma mitochondrial DNA DAMPs are linked to clinical outcome in severely injured human subjects. Ann Surg. (2013) 258:591–6. 10.1097/SLA.0b013e3182a4ea46 23979273 PMC3935616

[7] Jaber BL Pereira BJ. Extracorporeal adsorbent-based strategies in sepsis. Am J Kidney Dis. (1997) 30 (5 Suppl. 4), S44–56. 9372979 10.1016/s0272-6386(97)90542-4

[8] Rajaee A Barnett R Cheadle WG. Pathogen- and danger-associated molecular patterns and the cytokine response in sepsis. Surg Infect. (2018) 19:107–16. 10.1089/sur.2017.264 29364781

[9] Jansen MPB Pulskens WP Butter LM Florquin S Juffermans NP Roelofs J. Mitochondrial DNA is released in urine of SIRS patients with acute kidney injury and correlates with severity of renal dysfunction. Shock (2018) 49:301–10. 10.1097/SHK.0000000000000967 28837526

[10] Hu Q Ren J Ren H Wu J Wu X Liu S. Urinary mitochondrial DNA identifies renal dysfunction and mitochondrial damage in sepsis-induced acute kidney injury. Oxid Med Cell Longev. (2018) 2018:8074936. 10.1155/2018/8074936 29682165 PMC5846356

[11] He J Lu Y Xia H Liang Y Wang X Bao W Circulating mitochondrial DAMPs are not effective inducers of proteinuria and kidney injury in rodents. PLoS ONE (2015) 10:e0124469 10.1371/journal.pone.0124469 25902071 PMC4406729

[12] Tsuji N Tsuji T Ohashi N Kato A Fujigaki Y Yasuda H. Role of mitochondrial DNA in septic AKI via toll-like receptor 9. J Am Soc Nephrol. (2016) 27:2009–20. 10.1681/ASN.2015040376 26574043 PMC4926971

[13] Bellomo R Tipping P Boyce N. Continuous veno-venous hemofiltration with dialysis removes cytokines from the circulation of septic patients. Crit Care Med. (1993) 21:522–6. 8472571 10.1097/00003246-199304000-00011

[14] Piccinni P Dan M Barbacini S Carraro R Lieta E Marafon S. Early isovolaemic haemofiltration in oliguric patients with septic shock. Intensive Care Med. (2006) 32:80–6. 10.1007/s00134-005-2815-x 16328222

[15] Villa G Neri M Bellomo R Cerda J De Gaudio AR De Rosa S. Nomenclature for renal replacement therapy and blood purification techniques in critically ill patients: practical applications. Crit Care (2016) 20:283. 10.1186/s13054-016-1456-5 27719676 PMC5056485

[16] Park JT Lee H Kee YK Park S Oh HJ Han SH. High-dose versus conventional-dose continuous venovenous hemodiafiltration and patient and kidney survival and cytokine removal in sepsis-associated acute kidney injury: a randomized controlled trial. Am J Kidney Dis. (2016) 68:599–608. 10.1053/j.ajkd.2016.02.049 27084247

[17] Chung KK Coates EC Smith DJ Jr Karlnoski RA Hickerson WL Arnold-Ross AL. High-volume hemofiltration in adult burn patients with septic shock and acute kidney injury: a multicenter randomized controlled trial. Crit Care (2017) 21:289. 10.1186/s13054-017-1878-8 29178943 PMC5702112

[18] Ueno T Ikeda T Yokoyama T Kihara Y Konno O Nakamura Y. Reduction in circulating level of HMGB-1 following continuous renal replacement therapy in sepsis. Cytokine (2016) 83:206–9. 10.1016/j.cyto.2016.05.004 27155819

[19] Kellum JA Lameire N Group KAGW. Diagnosis, evaluation, and management of acute kidney injury: a KDIGO summary (Part 1). Crit Care (2013) 17:204. 10.1186/cc11454 23394211 PMC4057151

[20] Hu Q Ren J Wu J Li G Wu X Liu S. Elevated levels of plasma mitochondrial DNA are associated with clinical outcome in intra-abdominal infections caused by severe trauma. Surg Infect. (2017) 18:610–8. 10.1089/sur.2016.276 28414569

[21] Döcke WD Hoflich C Davis KA Rottgers K Meisel C Kiefer P. Monitoring temporary immunodepression by flow cytometric measurement of monocytic HLA-DR expression: a multicenter standardized study. Clin Chem. (2005) 51:2341–7. 10.1373/clinchem.2005.052639 16214828

[22] Perez-Fernandez X Sabater-Riera J Sileanu FE Vazquez-Reveron J Ballus-Noguera J Cardenas-Campos P. Clinical variables associated with poor outcome from sepsis-associated acute kidney injury and the relationship with timing of initiation of renal replacement therapy. J Crit Care (2017) 40:154–60. 10.1016/j.jcrc.2017.03.022 28407544

[23] Rhodes A Evans LE Alhazzani W Levy MM Antonelli M Ferrer R. Surviving sepsis campaign: international guidelines for management of sepsis and septic shock: 2016. Crit Care Med. (2017) 45:486–552. 10.1097/CCM.0000000000002255 28098591

[24] Pipili C Vasileiadis I Grapsa E Tripodaki ES Ioannidou S Papastylianou A. Microcirculatory alterations during continuous renal replacement therapy in ICU: a novel view on the 'dialysis trauma' concept. Microvasc Res. (2016) 103:14–8. 10.1016/j.mvr.2015.09.004 26431994

[25] Rosin DL Okusa MD. Dangers within: DAMP responses to damage and cell death in kidney disease. J Am Soc Nephrol. (2011) 22:416–25. 10.1681/ASN.2010040430 21335516 PMC4493973

[26] Xie L Liu S Cheng J Wang L Liu J Gong J. Exogenous administration of mitochondrial DNA promotes ischemia reperfusion injury via TLR9-p38 MAPK pathway. Regul Toxicol Pharmacol. (2017) 89:148–54. 10.1016/j.yrtph.2017.07.028 28757323

[27] Gouel-Cheron A Allaouchiche B Floccard B Rimmele T Monneret G. Early daily mHLA-DR monitoring predicts forthcoming sepsis in severe trauma patients. Intensive Care Med. (2015) 41:2229–30. 10.1007/s00134-015-4045-1 26359166

[28] Timmermans K Kox M Vaneker M van den Berg M John A van Laarhoven A. Plasma levels of danger-associated molecular patterns are associated with immune suppression in trauma patients. Intensive Care Med. (2016)42:551–61. 10.1007/s00134-015-4205-3 26912315 PMC5413532

[29] Monneret G Venet F. Sepsis-induced immune alterations monitoring by flow cytometry as a promising tool for individualized therapy. Cytometry B Clin Cytom. (2016) 90:376–86. 10.1002/cyto.b.21270 26130241

[30] Gainaru G Papadopoulos A Tsangaris I Lada M Giamarellos-Bourboulis EJ Pistiki A Increases in inflammatory and CD14(dim)/CD16(pos)/CD45(pos) patrolling monocytes in sepsis: correlation with final outcome. Crit Care (2018) 22:56 10.1186/s13054-018-1977-1 29499723 PMC5834896

[31] Li S Luo C Yin C Peng C Han R Zhou J. Endogenous HMGB1 is required in endotoxin tolerance. J Surg Res. (2013) 185:319–28. 10.1016/j.jss.2013.05.062 23866790

[32] Parker KH Sinha P Horn LA Clements VK Yang H Li J. HMGB1 enhances immune suppression by facilitating the differentiation and suppressive activity of myeloid-derived suppressor cells. Cancer Res. (2014) 74:5723–33. 10.1158/0008-5472.CAN-13-2347 25164013 PMC4199911

[33] Aneja R Odoms K Dunsmore K Shanley TP Wong HR. Extracellular heat shock protein-70 induces endotoxin tolerance in THP-1 cells. J Immunol. (2006) 177:7184–92. 10.4049/jimmunol.177.10.7184 17082636

[34] Borges TJ Wieten L van Herwijnen MJ Broere F van der Zee R Bonorino C. The anti-inflammatory mechanisms of Hsp70. Front Immunol. (2012) 3:95. 10.3389/fimmu.2012.00095 22566973 PMC3343630

[35] Asea A Kraeft SK Kurt-Jones EA Stevenson MA Chen LB Finberg RW. HSP70 stimulates cytokine production through a CD14-dependant pathway, demonstrating its dual role as a chaperone and cytokine. Nat Med. (2000) 6:435–42. 10.1038/74697 10742151

[36] Fong JJ Sreedhara K Deng L Varki NM Angata T Liu Q. Immunomodulatory activity of extracellular Hsp70 mediated via paired receptors Siglec-5 and Siglec-14. EMBO J. (2015) 34:2775–88. 10.15252/embj.201591407 26459514 PMC4682649

[37] Schafer ST Franken L Adamzik M Schumak B Scherag A Engler A. Mitochondrial DNA: an endogenous trigger for immune paralysis. Anesthesiology (2016) 124:923–33. 10.1097/ALN.0000000000001008 26808636

[38] Leijte GP Custers H Gerretsen J Heijne A Roth J Vogl T. Increased plasma levels of danger-associated molecular patterns are associated with immune suppression and postoperative infections in patients undergoing cytoreductive surgery and hyperthermic intraperitoneal chemotherapy. Front Immunol. (2018) 9:663. 10.3389/fimmu.2018.00663 29675023 PMC5895648

[39] Timmermans K Kox M Scheffer GJ Pickkers P. Plasma nuclear and mitochondrial DNA levels, and markers of inflammation, shock, and organ damage in patients with septic shock. Shock (2016) 45:607–12. 10.1097/SHK.0000000000000549 26717107

