# Supplementary figures and images for: Effects of Changes in the Levels of Damage-Associated Molecular Patterns Following Continuous Veno–Venous Hemofiltration Therapy on Outcomes in Acute Kidney Injury Patients With Sepsis
Source: Front Immunol. 2019 Jan 7;9:3052. doi: 10.3389/fimmu.2018.03052 (PMC6330765; doi:10.3389/fimmu.2018.03052)

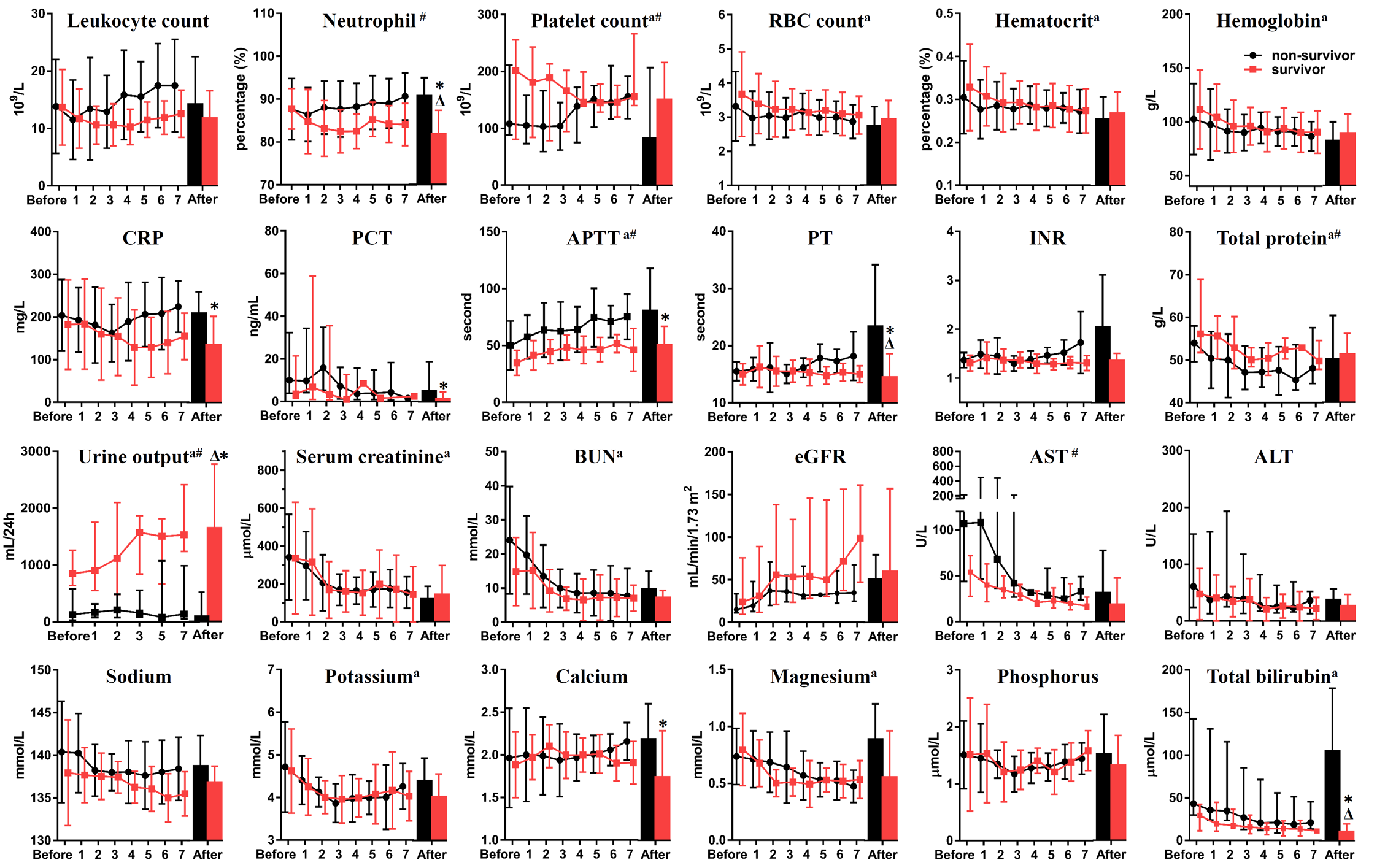

Supplement: Figure S1 — Tendency of change in clinical data under CVVH in survived and non-survived septic AKI patients. Twenty-four clinical indexes, including blood routine, liver function, renal function, electrolyte concentration, and coagulation function, etc. were collected every day during the first 7 days after CVVH initiation to form the line chart. The values at “After” denotes the indexes were acquired within 1 day of termination of CVVH treatment. aTendency of change was significant in total patients (P < 0.05). #Tendency was significant different between survived and non-survived sepsis patients (P < 0.05). ΔThe difference between before and after CVVH were significant distinct between survived and non-survived groups (P < 0.05). *The values were significant different between survived and non-survived groups after finished CVVH treatment (P < 0.05). BUN, blood urea nitrogen; eGFR, estimated glomerular filtration; ALT, Alanine aminotransferase; AST, Aspartate amino transferase; CRP, C-reaction protein; PCT, procalcitonin; RBC, red blood cell; PLT, platelet; APTT, activated partial thromboplastin time; PT, prothrombin time; INR, International Normalized Ratio. [file Image_1.TIF]

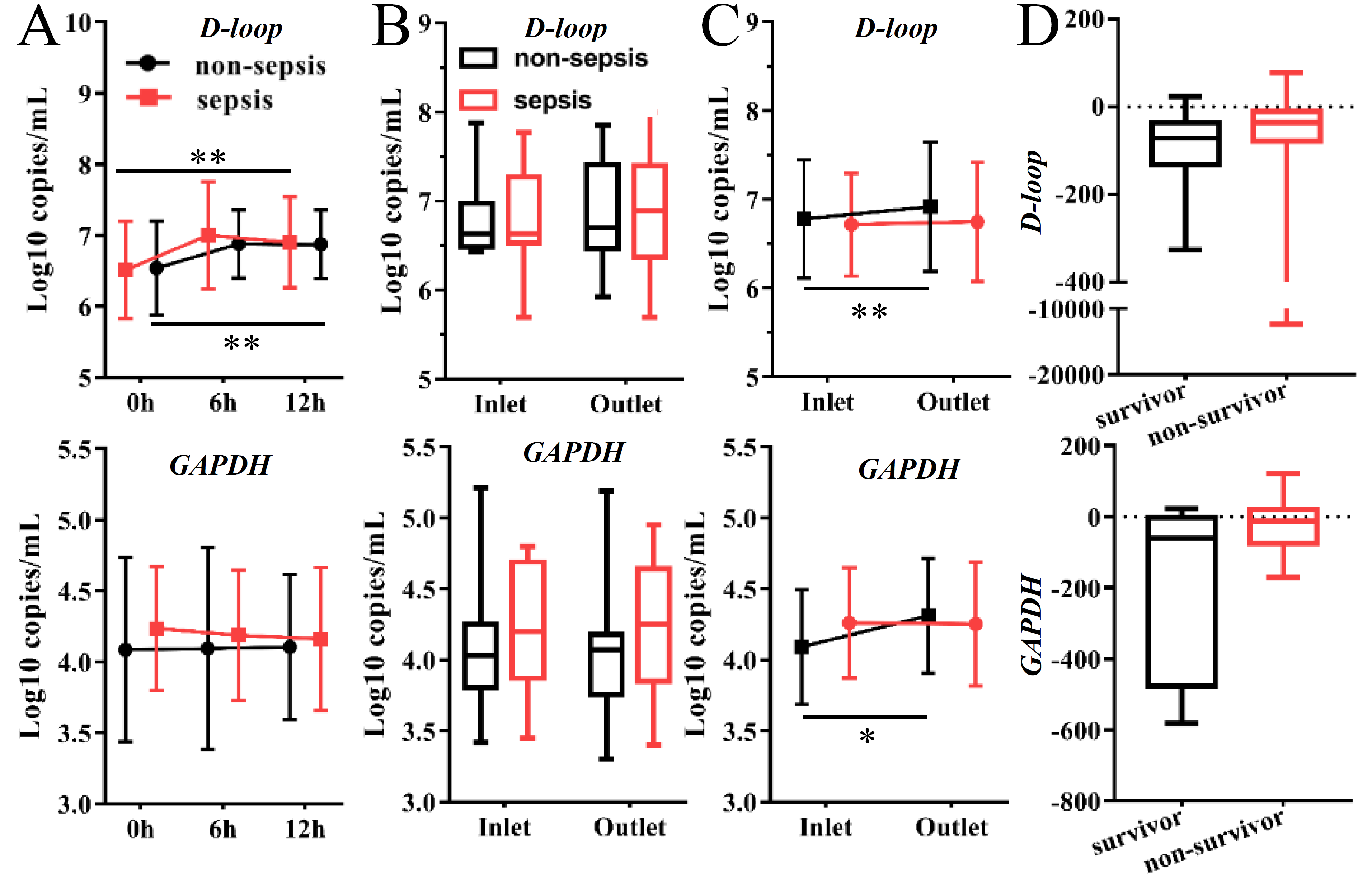

Supplement: Figure S2 — Effects of CVVH on D-loop (mtDNA) and GAPDH (nDNA) level and the clearance rate of them. Mitochondrial DNA (D-loop), nuclear DNA (GAPDH) were measured at baseline, 6, 12 h of CVVH at inlet and outlet. (A) Tendency of their levels during the first 12 h was analyzed by repeated measure ANOVA in sepsis and non-sepsis groups, respectively. Error bars of the line chart denote the mean with SD. (B) Box plots shown the levels of DAMPs at inlet and outlet in sepsis and non-sepsis groups. (C) Comparisons of mean levels of mtDNA and nDNA in survived or non-survived septic patients. (D) Box plots shown the levels of clearance rate (median ± IQR) in survived or non-survived septic patients. *P < 0.05, **P < 0.01, P < 0.001. DAMPs, damage-associated molecular patterns; mtDNA, mitochondrial DNA; nDNA, nuclear DNA; CVVH, continuous veno-venous hemofiltration; IQR, interquartile range. [file Image_2.TIF]
