# Supplementary material for: Effects of Changes in the Levels of Damage-Associated Molecular Patterns Following Continuous Veno–Venous Hemofiltration Therapy on Outcomes in Acute Kidney Injury Patients With Sepsis
Source: Front Immunol. 2019 Jan 7;9:3052. doi: 10.3389/fimmu.2018.03052 (PMC6330765; doi:10.3389/fimmu.2018.03052)
Supplement: Table S1 — Receiver Operating Characteristic curves (ROC) of the clearance of DAMPs and cytokines to predict hospital mortality. Fold front denotes the significance of ROC analysis was <0.05. The cut-off values and corresponding sensitivity and specificity were calculated based on Youden index. IFN, interferon; IL, interleukin; TNF, tumor necrosis factor; DAMPs, Damage-Associated Molecular Patterns; mtDNA, mitochondrial DNA; nDNA, nuclear DNA; HSP70, Heat Shock Protein 70; HMGB1, high-mobility group box 1. [file Table_1.DOCX]

**Supplementary Table 1. ROC analysis to assess the prediction factors for mortality in septic patients.**

| **Variables** | | **AUC (95%CI)** | ***P* value** | **Cut-off point** | **Sensitivity** | **Specificity** |
| --- | --- | --- | --- | --- | --- | --- |
| **mCL_cytokines_** | |  |  |  |  |  |
| IL-1b | | 0.68 (0.47-0.88) | 0.125 |  |  |  |
| IL-6 | | 0.35 (0.14-0.57) | 0.195 |  |  |  |
| IFN-γ | | 0.63 (0.40-0.87) | 0.250 |  |  |  |
| TNF-α | | 0.32 (0.05-0.58) | 0.114 |  |  |  |
| IL-10 | | 0.55 (0.31-0.79) | 0.666 |  |  |  |
| **mCL_DAMPs_** | |  |  |  |  |  |
| HSP70 | | **0.93 (0.84-1.00)** | **0.000** | **-0.49** | **0.78** | **1.00** |
| *ND2* | | 0.58 | 0.472 |  |  |  |
| *D-loop* | | 0.60 | 0.388 |  |  |  |
| *GAPDH* | | 0.66 | 0.164 |  |  |  |
| *β-globin* | | **0.77 (0.54-1.00)** | **0.017** | **-118.59** | **0.94** | **0.80** |
| **mC_DAMPs_** | |  |  |  |  |  |
| HSP70, ng/mL | inlet | **0.73 (0.54-0.93)** | **0.044** | **100.82** | **0.56** | **1.00** |
|  | outlet | 0.72 (0.52-0.91) | 0.061 |  |  |  |
| *ND2*,  log10 copies/mL | inlet | 0.51 (0.29-0.73) | 0.156 |  |  |  |
|  | outlet | 0.48 (0.25-0.71) | 0.615 |  |  |  |
| *D-loop*,  log10 copies/mL | inlet | 0.53 (0.29-0.77) | 0.973 |  |  |  |
|  | outlet | 0.48 (0.25-0.71) | 0.623 |  |  |  |
| *GAPDH*,  log10 copies/mL | inlet | **0.54 (0.30-0.77)** | **0.001** | **4.44** | **0.33** | **0.90** |
|  | outlet | 0.44 (0.21-0.66) | 0.109 |  |  |  |
| *β-globin*,  log10 copies/mL | inlet | **0.75 (0.57-0.93)** | **0.000** | **3.97** | **0.50** | **1.00** |
|  | outlet | **0.48 (0.22-0.74)** | **0.034** | **3.49** | **0.89** | **0.40** |

Abbreviations: *mCL_cytokines_* the mean of cytokines clearance rate, *mCL_DAMPs_* the mean of DAMPs clearance rate, mC_DAMPs,_ mean of circulating DAMPs levels, *DAMPs* Damage-Associated Molecular Patterns
